# Distribution of Total Depressive Symptoms Scores and Each Depressive Symptom Item in a Sample of Japanese Employees

**DOI:** 10.1371/journal.pone.0147577

**Published:** 2016-01-26

**Authors:** Shinichiro Tomitaka, Yohei Kawasaki, Kazuki Ide, Hiroshi Yamada, Hirotsugu Miyake, Toshiaki A. Furukaw

**Affiliations:** 1 Department of Mental Health, Panasonic Health Center, Tokyo, Japan; 2 Department of Drug Evaluation and Informatics, Graduate School of Pharmaceutical Sciences, University of Shizuoka, Shizuoka, Japan; 3 Hokkaido Occupational Health Promotion Center, Labor Health and Welfare, Sapporo, Japan; 4 Department of Health Promotion and Human Behavior, Kyoto University Graduate School of Medicine/School of Public Health, Kyoto, Japan; Chiba University Center for Forensic Mental Health, JAPAN

## Abstract

**Background:**

In a previous study, we reported that the distribution of total depressive symptoms scores according to the Center for Epidemiologic Studies Depression Scale (CES-D) in a general population is stable throughout middle adulthood and follows an exponential pattern except for at the lowest end of the symptom score. Furthermore, the individual distributions of 16 negative symptom items of the CES-D exhibit a common mathematical pattern. To confirm the reproducibility of these findings, we investigated the distribution of total depressive symptoms scores and 16 negative symptom items in a sample of Japanese employees.

**Methods:**

We analyzed 7624 employees aged 20–59 years who had participated in the Northern Japan Occupational Health Promotion Centers Collaboration Study for Mental Health. Depressive symptoms were assessed using the CES-D. The CES-D contains 20 items, each of which is scored in four grades: “rarely,” “some,” “much,” and “most of the time.” The descriptive statistics and frequency curves of the distributions were then compared according to age group.

**Results:**

The distribution of total depressive symptoms scores appeared to be stable from 30–59 years. The right tail of the distribution for ages 30–59 years exhibited a linear pattern with a log-normal scale. The distributions of the 16 individual negative symptom items of the CES-D exhibited a common mathematical pattern which displayed different distributions with a boundary at “some.” The distributions of the 16 negative symptom items from “some” to “most” followed a linear pattern with a log-normal scale.

**Conclusions:**

The distributions of the total depressive symptoms scores and individual negative symptom items in a Japanese occupational setting show the same patterns as those observed in a general population. These results show that the specific mathematical patterns of the distributions of total depressive symptoms scores and individual negative symptom items can be reproduced in an occupational population.

## Introduction

Depression is a common mental disorder that is among the leading causes of disability worldwide [1]. Given that depressive symptoms are closely linked with depression, there has been a great interest in understanding the distribution of depressive symptoms in the general population [2, 3]. However, despite the accumulation of knowledge regarding the prevalence of depressive symptoms, no mathematical model has been developed to explain the distribution of depressive symptoms in a population. Recently, our group reported three findings that may contribute to the development of a mathematical model capable of describing the distribution of depressive symptoms [4, 5].

First, the distribution of the Center for Epidemiologic Studies Depression Scale (CES-D) scores for total depressive symptoms in a general population remained stable from 30–69 years [4]. In agreement with our study, a number of other studies have reported that the average score for total depressive symptoms stabilizes during ages 30–69 years [6, 7]. The estimated average change per decade in the total CES-D score is less than 1 point between the ages of 30 and 69 years, whereas the standard deviation of CES-D scores in community surveys is between 5 to 10 points [4, 7]. To date, little attention has been given to the stability of the distribution of depressive symptoms according to age. In general, however, the distributions of several physiological parameters that exhibit individual variations, including blood pressure, blood glucose level, basal metabolic rate, and body weight, change considerably with age [8, 9, 10, 11]. The homeostasis of these various physiological parameters is not strong enough to stabilize their distributions over a period of many years. Taking the variability of these distributions of physiological parameters into account, the stability of the distribution of depressive symptoms according to age seems notable.

Second, both Meltzer et al. and our group have noted that the total depressive symptoms score follows an exponential curve except for at the lowest end of the symptoms score. Meltzer et al. reported that the total neurotic symptoms score and the depressive scores as evaluated using the Revised Clinical Interview Schedule (CIS-R), which consists of 14 negative symptom items (somatic symptoms, fatigue, concentration, sleep, irritability, worry about physical health, depression, depressive ideas, worry, anxiety, phobias, panic, compulsion, and obsession), follow exponential curves for symptom scores of more than 3 [12]. We previously reported that the total depressive symptoms score follows an exponential curve within the range of CES-D scores over 11 points [4]. These results indicate that the distribution of the total depressive symptoms score follows an exponential curve except for at the lowest end of the symptoms score.

Third, we showed that each item of the CES-D follows a specific pattern [5]. In the CES-D, each item has one question which is scored based on four possible answers: “rarely or none of the time (rarely),” “some or a small amount of time (some),” “occasionally or a moderate amount of time (much),” and “most of the time (most)” [13]. We have shown that the individual distributions of 16 negative items belonging to the depressive mood, somatic symptoms and retarded activities, and interpersonal relations categories commonly exhibited exponential patterns between “some” and “most” response levels, whereas “rarely” was the complement event of “some,” “much,” or “most” and was not related to the exponential patterns between “some” and “most.”

The degree to which these three findings can be generalized to other populations remains unclear. To the best of our knowledge, no report has investigated the reproducibility of these three findings in other populations. A statistical model would be useful to evaluate the depressive symptom scores of the individuals in a population.

The present study used some of the data from the Northern Japan Occupational Health Promotion Centers Collaboration Study for Mental Health (NOCS-MH), which was conducted by the Japanese Occupational Health Promotion Center to obtain data applicable to the promotion of occupational health [14]. Using more than 7,000 CES-D assessments performed in Japanese employees, we examined whether the distribution of the total depressive symptoms score of the CES-D remains stable throughout middle adulthood and whether it follows an exponential curve outside of the lowest end of the symptoms score. In addition, we also assessed whether 16 individual negative symptom items also follow exponential patterns between “some” and “most” response levels in a novel study population.

## Methods

### Measures

The present study used the Japanese version of the CES-D to evaluate depressive symptoms [15]. This 20-item scale assesses the frequency of a variety of depressive symptoms within the previous week (0 = rarely or none of the time, 1 = some of the time, 2 = much of the time, and 3 = most or all of the time), yielding a total score ranging from 0 to 60. Higher values indicate greater psychological distress. The positive affect items were reverse-scored.

The 20 items of the CES-D were grouped into the following 4 subscales: depressive mood (items 3, 6, 9, 10, 14, 17, and 18), somatic and retarded activities symptoms (items 1, 2, 5, 7, 11, 13, and 20), interpersonal relations (items 15 and 19), and positive affects (items 4, 8, 12, and 16).

### Data set and analysis procedure

Data from the Northern Japan Occupational Health Promotion Centers Collaboration Study for Mental Health (NOCS-MH), conducted by the Northern Japan Occupational Health Promotion Centers in 2010, were utilized. The NOCS-MH is a survey conducted by the Japanese Incorporated Administrative Agency, Occupational Health Promotion Center, to obtain data required for occupational health promotion. In 2010, the NOCS-MH examined depressive symptoms among a sample of Japanese employees. The NOCS-MH was approved by the ethics committee of the Japanese Incorporated Administrative Agency, Occupational Health Promotion Center (approval number: 2010–10). Survey participants were selected from individuals aged 15 years and over working for 103 companies across northern Japan (Hokkaido prefecture, Aomori prefecture, Iwate prefecture, Miyagi prefecture, Akita prefecture, Yamagata prefecture, and Fukushima prefecture). Because the questionnaires were filled out anonymously, written informed consent was not obtained. Oral informed consent was obtained from all of the subjects. The questionnaire was returned by 8,246 respondents. The response rate was 93.4%. The data and methods used by the survey have been published in detail [14].

We used some of the data from the NOCS-MH. Because the present study was focused on middle adulthood, our sample consisted of 7,624 respondents between the ages of 20–59 years (ages 20–29; N = 1661 [female; n = 708], ages 30–39; N = 2168 [female; n = 828], ages 40–49; N = 1978 [female; n = 802]; ages 50–59; N = 1816 [female; n = 707]). Our present study was approved by the ethics committee of the Panasonic Health Center (approval 2014–1).

### Statistical analysis

The respondents were grouped into the following age groups: 20–29 years, 30–39 years, 40–49 years, and 50–59 years. Descriptive statistics, such as the mean, standard deviation, skewness, kurtosis, and frequency curve, were calculated for each age group. To estimate the proportion of high CES-D scores, we calculated the 90th percentile of the CES-D score for each group.

The first step in this analysis was to ascertain whether the distribution of the total depressive symptoms scores as assessed using the CES-D was stable throughout middle adulthood and whether it followed an exponential pattern over a specific level of total depressive symptom scores. We compared the frequency curves among middle adulthood groups (30–39 years, 40–49 years, and 50–59 years). Subjects who did not respond to all the CES-D items were excluded (N = 639). Overall, 91.6% of the subjects responded to all the items.

After confirming that the distribution of the middle adulthood groups was stable, the right tails of the distributions for middle adulthood were analyzed using a log-normal scale to ascertain the exponential patterns of the frequency of total depressive symptoms during middle adulthood.

The next step was to examine whether the individual distributions of 16 negative items belonging to the 3 CES-D subscales for depressive mood, somatic symptoms and retarded activities, and interpersonal relations exhibited exponential patterns outside of the lowest end of the item score. Subjects who did not respond to each item were excluded from the percentage analysis. The response rate for each item varied from 96.1% to 99.1%. The item response percentages for the 16 negative items were analyzed using a normal scale and a log-normal scale.

We used JMP version 11 for Windows (SAS Institute, Inc., Cary, NC, USA) to calculate the descriptive statistics and the frequency distribution curves.

## Results

### Total CES-D score analysis

The descriptive statistics for the distributions of total CES-D scores according to age groups are shown in Table 1. The means of total depressive symptoms scores during ages 20–59 years exhibited a pattern of gradual reduction with age, with higher depressive symptoms scores during the 20–29 year age and then decreasing during middle age. Furthermore, the skewness, kurtosis, median and 90th percentile of the CES-D scores were similar among the middle age groups (30–39 years, 40–49 years, and 50–59 years).

**Table 1:** Summary of descriptive statistics for the total CES-D score distributions according to age group in a general Japanese population.

Age group	N	Mean	Skewness	Kurtosis	Median	90th percentile
20–29	1579	16.7 ± 8.4	0.79	0.88	16	28
30–39	2043	15.0 ± 8.2	0.85	1.03	14	26
40–49	1809	14.8 ± 8.3	0.98	1.50	13	26
50–59	1554	13.8 ± 7.7	0.90	1.10	13	25
Total	6985	15.1 ± 8.2	0.88	1.12	14	26

**Fig 1:**
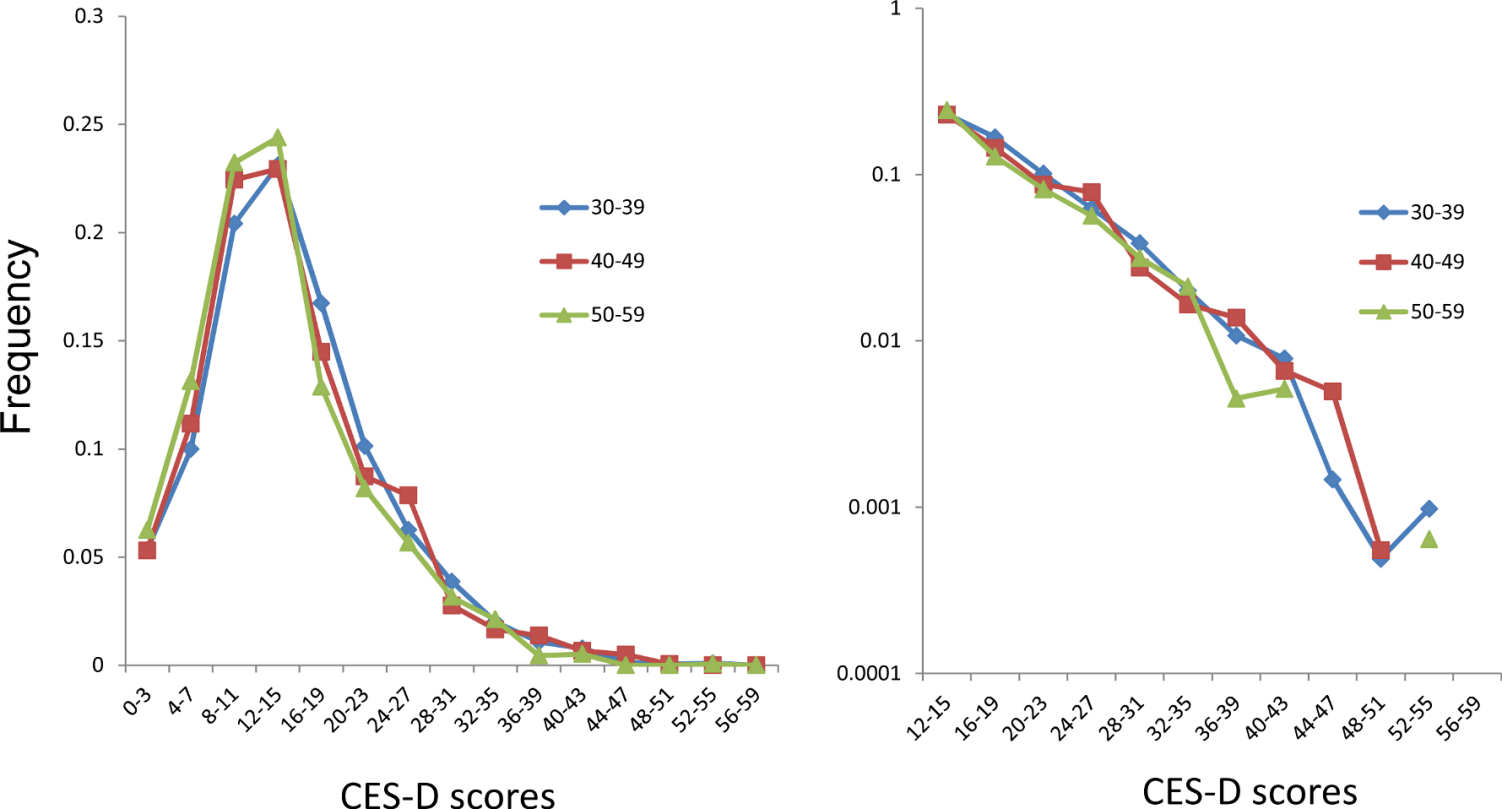
CES-D distributions for the middle adulthood groups (left). The right tails of the distributions for middle adulthood were compared using a log-normal scale (right).

Next, the distributions of the total CES-D scores were compared among the middle age groups (30–39 years, 40–49 years, and 50–59 years). Fig 1A shows that the distributions of the CES-D scores were similar to each other among the middle adulthood groups. Despite a 30-year range in age, the distribution of the CES-D score was stable throughout middle adulthood.

To ascertain the exponential patterns of the frequency of depressive symptoms during middle adulthood, the right tails of the distributions for middle adulthood were compared using a log-normal scale (Fig 1B). In the range of CES-D scores from 12–15 points to 40–43 points, the curves for each age group exhibited a linear pattern. However, for CES-D scores over 40–43 points, the curves for each age group fluctuated randomly, likely reflecting the small sample sizes for each age group. In support of the small sample sizes being a cause of the fluctuations, the percentage of CES-D scores over 40–43 points was sometimes zero for individual age groups (the absence of a data point in the line charts corresponds to a value of zero in Fig 1).

### Analysis of the distributions of the 16 individual negative symptom items

Table 2 shows the response rates for the 20 items in the CES-D questionnaire. The 16 negative items for the depressive mood subscale, the somatic symptoms and retarded activities subscale, and the interpersonal relations subscale exhibited a common pattern, with the highest response frequency for “rarely,” a decreasing response frequency as the item score increased, and the lowest response frequency observed for “most.” No exceptions to this pattern were observed. The 4 positive items in the positive affect subscale did not exhibit a specific pattern.

**Table 2:** Item responses of a sample of Japanese employees.

Number	Item/Subscale	Response (%)			
	Depressed Mood	Rarely	Some	Much	Most
3	Blues	4418 (58.7)	1951 (25.9)	777 (10.3)	376 (5.0)
6	Depressed	3069 (40.6)	2520 (33.3)	1323 (17.5)	652 (8.6)
9	Failure	2695 (36.8)	2724 (37.2)	1292 (17.7)	605 (8.3)
10	Fearful	5436 (71.8)	1288 (17.0)	596 (7.9)	253 (3.3)
14	Lonely	5228 (69.0)	1391 (18.4)	609 (8.0)	350 (4.6)
17	Crying	6725 (88.8)	566 (7.5)	204 (2.7)	81 (1.1)
18	Sad	4925 (65.4)	1810 (24.0)	559 (7.4)	242 (3.2)
	**Somatic symptoms and retarded activities**				
1	Bothered	3014 (39.8)	3074 (40.6)	1155 (15.3)	330 (4.4)
2	Appetite	5486 (72.3)	1381 (18.2)	583 (7.7)	142 (1.9)
5	Trouble concentrating	2991 (39.5)	2882 (38.1)	1366 (18.1)	327 (4.3)
7	Effort	2623 (34.7)	2986 (39.5)	1284 (17.0)	658 (8.7)
11	Sleep	4631 (61.2)	1791 (23.7)	777 (10.3)	366 (4.8)
13	Talked	3823 (50.5)	2314 (30.6)	952 (12.6)	479 (6.3)
20	Get going	5212 (68.8)	1747 (23.1)	478 (6.3)	134 (1.8)
	**Interpersonal relations**				
15	Unfriendly	5232 (69.3)	1522 (20.1)	584 (7.7)	217 (2.9)
19	Dislike	5074 (67.0)	1784 (23.6)	500 (6.6)	215 (2.8)
	**Positive affects**				
4	Good	1924 (26.1)	2299 (31.2)	1540 (20.9)	1615 (21.9)
8	Hopeful	2193 (29.1)	2766 (36.8)	1646 (21.9)	921 (12.2)
12	Happy	1765 (23.4)	2639 (35.0)	1633 (21.7)	1504 (19.9)
16	Enjoyed	1739 (23.1)	2596 (34.4)	2172 (28.8)	1033 (13.7)

**Fig 2:**
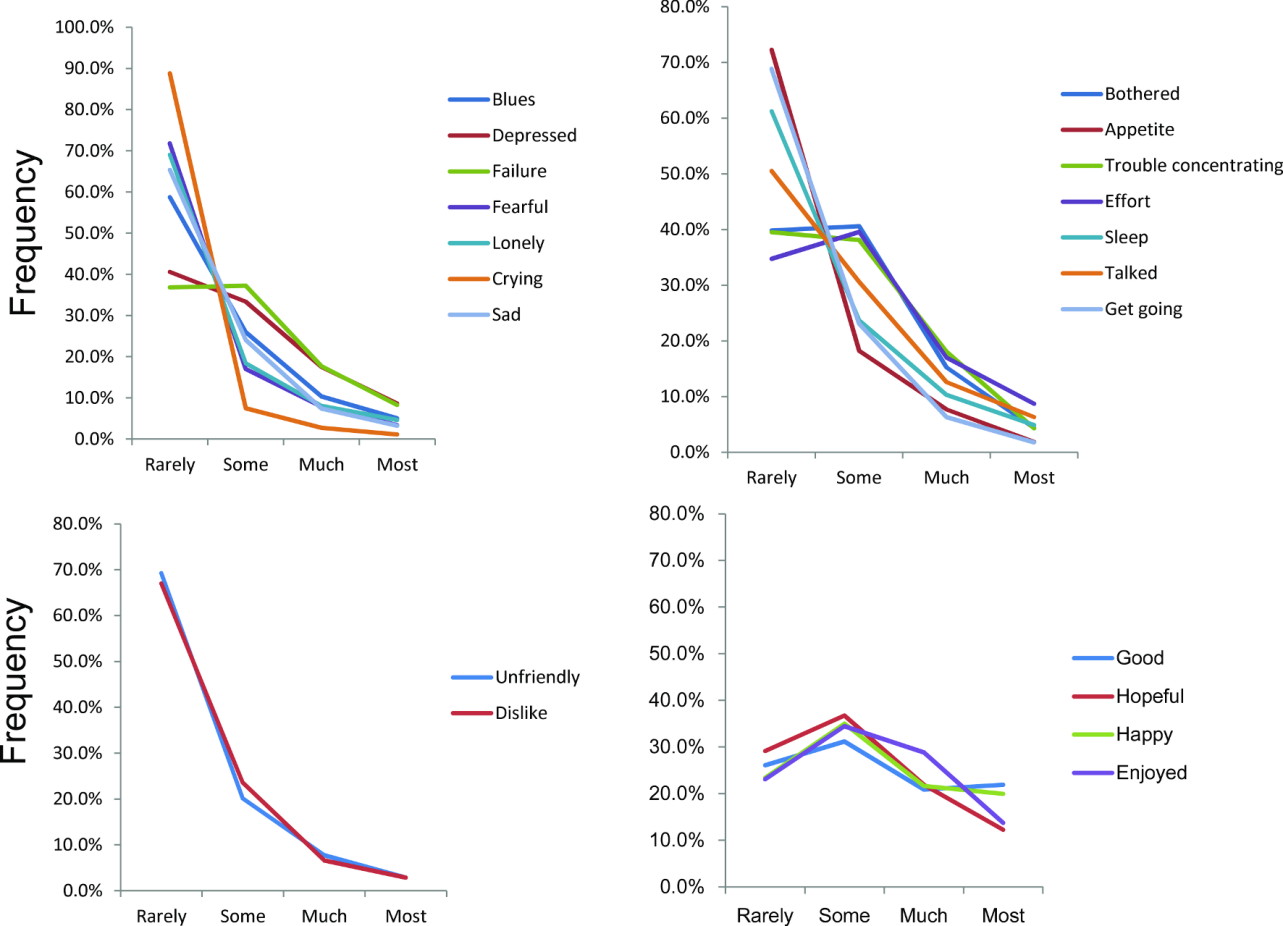
Distributions of the item responses for the depressive mood group (upper left), the somatic symptoms and retarded activities group (upper right), the interpersonal relations group (bottom left), and the positive affect group (bottom right).

As depicted in Fig 2, the items of the depressive mood subscale (2A), the somatic symptoms and retarded activities subscale (2B) and the interpersonal relations subscale (2C), exhibited right-skewed distributions in common, whereas the 4 items of the positive affect subscale (2D) exhibited non-specific, reverse U-shaped distributions, suggesting that the distribution pattern of the positive affect subscale differed from those of the other groups.

**Fig 3:**
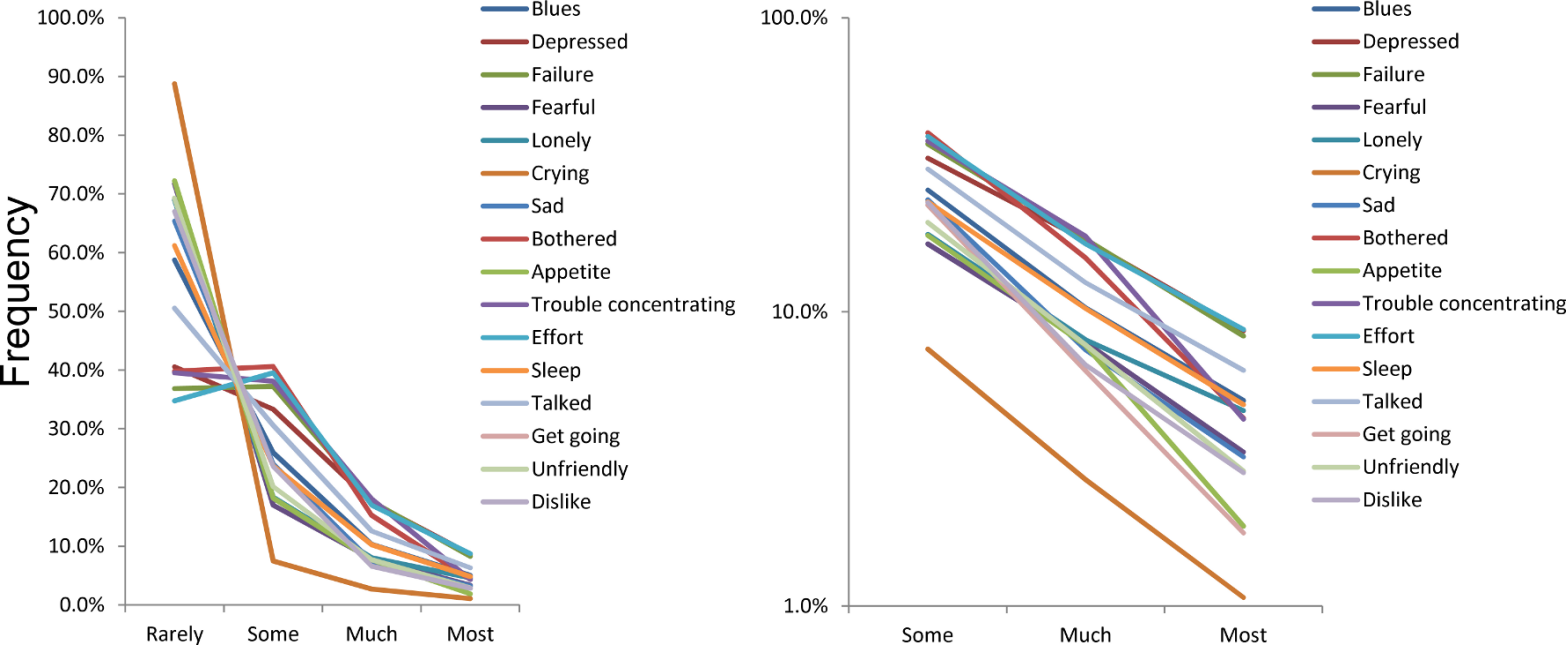
The distributions of the 16 negative items were compared using a normal scale (left) and a log-normal scale (right).

To evaluate the distributions of the 16 individual negative symptom items, the distributions were plotted together on the same graph. As depicted in Fig 3A, the distributions of each of the 16 negative items had different patterns, with a boundary at “some” between “rarely” and “some”. The lines for the 16 items crossed each other, whereas the lines from “some” to “most” showed a pattern of reduction.

As verified in our previous study, if the distributions of the 16 items follow an exponential pattern between “some” and “most” with the same parameter, all the lines for the 16 items must inevitably cross at a single point between “rarely” and “some” [5]. Although the crossings did not necessarily occur at a single point between “some” and “most,” the lines for the 16 negative items appeared to cross in the vicinity of a single point.

Using a log-normal scale, the distributions of the 16 items showed a linear pattern for the “some” to “most” responses (Fig 3B). In addition, the lines for the 16 items were almost parallel to each other, suggesting that the gradients of the linear patterns for the 16 items were similar to each other. However, the lines for several items, including “Appetite,” “Get going,” “Bothered,” and “Trouble concentrating” were not parallel at the “most” level.

## Discussion

The results of this study indicate that the distribution of total depressive symptoms scores in a Japanese occupational setting is stable throughout middle adulthood and displays an exponential pattern except for at the lowest end of the symptom score. Furthermore, the present study shows that the distribution of individual negative symptom items exhibits an exponential pattern from “some” to “most,” although the degree of fit with the exponential pattern from “some” to “most” differed according to the negative item. These findings are consistent with previous studies performed in a general population [4, 5]. These results indicate that the three findings reported in our previous report can be reproduced in a Japanese occupational population.

In the present study, the distribution of total depressive symptoms scores was stable throughout middle adulthood. The reason why the distribution of depressive symptoms is stable during middle adulthood is unknown. One possibility is that individual homeostasis during middle adulthood is strong enough to stabilize the distributions of depressive symptoms in the general population. However, this explanation conflicts with the fact that people are most likely to suffer their first depressive episode between the ages of 30 and 60 years [16]. Although the mechanism is unknown, social homeostasis of depressive symptoms is sustained in some way. Of note, an exponential distribution is observed in situations in which individual variability and total stability are organized together, such as in the Bolzman-Gibbs law, atmospheric pressure, and money distribution [17].

In the range of CES-D scores from 12–15 points to 40–43 points, the distributions for each age group exhibited a linear pattern with a log-normal distribution. With respect to the range of the exponential pattern, the finding of the present study concurs with that of the previous study performed in a general population [4]. The range of the exponential pattern is noteworthy because the range covers clinical depression. A score of 25 is frequently defined as the threshold for depression in Eastern Asia, whereas a score of 16 is typically considered to be the threshold for clinical depression in Western countries [18]. Our results indicate that the exponential pattern covers a wide range of CES-D scores beyond the threshold for depression.

Consistent with a previous report [5], the distributions for each of the 16 negative items displayed different patterns, with a boundary at “some.” The mechanism responsible for the different patterns with a boundary at “some” could be explained as follows. In general, self-rating depression scales are scored using a two-step process. First, each subject must determine whether a symptom is present. If the level of the symptom does not meet the threshold that excludes everyday mild symptoms, it is regarded as “rarely.” Next, if a depressive symptom that meets the threshold is present, the duration of the symptom is quantified and divided into “some,” “much,” and “most.” This two-step process increases the possibility that “rarely” will cover the range that does not meet the threshold, while each of “some,” “much,” and “most” will cover about one-third of the range that satisfies the threshold. If the range for “rarely” differs from those of “some,” “much” and “most,” the distributions of the 16 negative items will display different patterns with a boundary at “some.” Further consideration regarding this speculation is needed.

In the present study, the 16 individual negative symptom items followed an exponential pattern with the same parameter between “some” and “most.” However, the degree to which the 16 individual negative symptom items followed an exponential pattern with the same parameter was not very strong compared with the results of our previous general population sample (N = 32022) [5]. The lines for several items, including “Appetite,” “Get going,” “Bothered,” and “Trouble concentrating” were not parallel at the “most” level, whereas most of the lines for the 16 items were parallel to each other (Fig 3A). In support of these findings, the lines for the 16 negative items appeared to cross not at a single point, but in the vicinity of a single point (Fig 3B). As verified in our previous study, all the lines that follow an exponential pattern with the same parameter between “some” and “most” have to cross at a single point between “rarely” and “some” [5]. The reason for the difference in the reproducibility of the exponential pattern from “some” to “most” may be related to the selection bias. The subjects in the previous general population, which displayed a distinct exponential pattern from “some” to “most,” were randomly recruited to be representative of the Japanese general population using a stratified sampling design, whereas the subjects for this study were non-randomly selected from particular workplaces.

Although the distributions of the four positive affects appeared in a reverse U-shaped distribution in the present study, the evidence for the response patterns of positive affect symptoms has been mixed. A number of cross-cultural comparison studies have reported that the response patterns for the positive affect items vary according to ethnicity or nation (e.g., skewed, plateau-shaped, U-shaped and reverse U-shaped), whereas the response patterns for the 16 negative items are generally comparable [19, 20]. Recently, positive affect and negative affect have been recognized as two different phenomena that should be studied individually [21, 22]. Our results lend credibility to the view that positive affect and negative affect are two different phenomena.

The present study has some limitations. First, a standard psychiatric diagnosis was not performed for the subjects. Therefore, the study did not encompass a diagnosis of depressive symptoms. Second, while our data collection procedure attempted to obtain a representative group of employees by selecting 7624 employees of 103 companies, the selection of companies as a base for data collection limits the population to which our findings may be generalized. Finally, although we examined the exponential pattern using a log-normal scale, analysis based on other mathematical models was not performed.

Despite these limitations, the present study shows that the specific mathematical patterns of the distributions of total depressive symptoms scores and individual negative symptom items can be reproduced in a sample of Japanese employees. A statistical model is useful to evaluate the scores of the individuals in a population. The present study could provide a statistical model for a self-report depressive symptoms questionnaire in an occupational population. The degree to which these findings can be generalized to other populations or other scales for depressive symptoms is unclear but warrants examination.
