# Correction: Distribution of Total Depressive Symptoms Scores and Each Depressive Symptom Item in a Sample of Japanese Employees

**DOI:** 10.1371/journal.pone.0152645

**Published:** 2016-03-24

**Authors:** Shinichiro Tomitaka, Yohei Kawasaki, Kazuki Ide, Hiroshi Yamada, Hirotsugu Miyake, Toshiaki A. Furukawa

The sixth author’s name is incorrectly spelled. The correct name is: Toshiaki A. Furukawa. The correct citation is: Tomitaka S, Kawasaki Y, Ide K, Yamada H, Miyake H, Furukawa TA (2016) Distribution of Total Depressive Symptoms Scores and Each Depressive Symptom Item in a Sample of Japanese Employees. PLoS ONE 11(1): e0147577. doi:10.1371/journal.pone.0147577
